# Quantitative Assessment of Outer Retinal Layer and Photoreceptor Outer Segment Layer and Their Relation to Visual Acuity in Diabetic Macular Edema

**DOI:** 10.1155/2019/8216150

**Published:** 2019-02-17

**Authors:** Ahmed Hosni Abd Elhamid

**Affiliations:** ^1^Assistant Professor of Ophthalmology, Ain Shams University, Cairo, Egypt; ^2^Vitreoretinal Consultant, Hadi Hospital, Jabriya, Kuwait

## Abstract

**Purpose:**

To compare outer retinal layer (ORL) thickness and photoreceptor outer segment (PROS) length between normal eyes and eyes with diabetic macular edema (DME), and also, to study the correlation between central macular thicknesses (CMT), ORL, and PROS length with best corrected visual acuity (BCVA) in DME.

**Methods:**

80 eyes were included in the study; they were divided into two groups, group Ι (40 normal eyes) and group ΙΙ (40 eyes) with DME. Complete ophthalmic examination and OCT were done for all eyes. Comparison between ORL and PROS was done between both groups; also, correlation between ORL thickness, PROS length, and CMT with BCVA in group ΙΙ was studied.

**Results:**

CMT was greater in group ΙΙ than group Ι (392.70 ± 62.91 and 265.73 ± 17.17, respectively) (SS, *p* < 0.001). ORL thickness was statistically significantly greater in group Ι than group ΙΙ (104.80 ± 4.94, 93.68 ± 6.34, *p* < 0.001). Regarding PROS length, it was statistically significantly greater in group Ι than group ΙΙ (31.38 ± 3.4 and 26.65 ± 3.39, respectively, *p* < 0.001). There was moderate correlation between BCVA and ORL thickness (*r* = −0.440, *p*=0.004) and strong correlation between BCVA and PROS length in group ΙΙ (*r* = − 0.690, *p* < 0.001), while there was weak correlation between BCVA and CMT (*r* = 0.198, *p*=0.220).

**Conclusion:**

Both ORL thickness and PROS length were greater in healthy normal eyes than eyes with DME. BCVA was correlated better with PROS length and ORL thickness than CMT.

## 1. Introduction

Diabetic macular edema (DME) is a leading cause of visual loss [1]. Pathological retinal abnormalities in patients with diabetic retinopathy are subject for research and clinical studies since long time [2].

Optical coherence tomography (OCT) is a reliable noncontact and noninvasive diagnostic tool used in many retinal disorders including DME. Recent OCT machines with fast scanning speed and high axial resolution which reaches up to three microns enable retinal specialists to identify and study retinal microstructures like retinal pigmented epithelium (RPE), external limiting membrane (ELM), and inner segment/outer segment junction (IS/OS) [3]. The most common objective parameter used in assessment of DME patients is central macular thickness (CMT). Many studies showed contradicting correlation between the measured CMT and best corrected visual acuity (BCVA) in DME [4–7].

Photoreceptor is the basic cell that is responsible for signal transmission in the retina, so it has been studied whether it could be affected or not in DME. In patients with diabetes and no retinopathy, the photoreceptor cell layer at the fovea was found to be thinner than the corresponding layer in healthy volunteers. In patients with DME, BCVA was found to vary according to PROS thickness, particularly when this thickness was measured at the center of the fovea [8]. The integrity of the IS/OS junction has been found to correlate well with visual acuity in subjects with retinal diseases such as retinitis pigmentosa and postmacular hole surgery [3].

External limiting membrane (ELM) is another important retinal structure. Being situated between the cell nucleus and inner segments of photoreceptors, ELM may also be a possible OCT-based parameter to be used indirectly in the assessment of photoreceptor functions [9].

Both IS/OS integrity and PROS have been reported to be indicators of BCVA in DME and other retinal diseases [10]. Forooghian et al. measured the distance between the IS/OS junction and RPE layer (PROS) and showed that vision correlated better with PROS length than CMT in DME [11].

The aim of this study is to compare outer retinal layer thickness and photoreceptor outer segment length between normal eyes and eyes with diabetic macular edema and also to study the correlation between CMT, ORL thickness, and PROS length with BCVA in DME.

## 2. Methods

Prospective, nonrandomized observational comparative study was conducted and included 80 eyes of 55 patients. The study was carried out in retina clinic, Ophthalmology Department, Hadi Hospital in Kuwait, during the period from January 2017 till October 2017. Inclusion criteria are age from 55 to 70 years, any sex, and patients with center-involved diabetic macular edema diagnosed clinically and confirmed by OCT with CMT more than 300 *µ*m are included in group ΙΙ. Exclusion criteria are significant media opacity that can lead to poor quality and signal of the OCT image; other conditions that are excluded from group ΙΙ are neurosensory detachment, previous laser treatment, subfoveal exudates, and disrupted ELM or IS\OS junction. Also, any condition that can lead to macular edema rather than diabetic retinopathy like epiretinal membrane, age-related macular degeneration, and retinal vascular occlusive diseases are excluded.

The study was performed in accordance with the Declaration of Helsinki. The eyes are divided into two groups: group Ι included normal 40 healthy eyes of 20 volunteers and group ΙΙ that included 40 eyes with DME from 35 patients.

All eyes had undergone complete ophthalmic examination including BCVA measured by the Snellen acuity chart, IOP measured by Goldmann tonometry, and dilated fundus examination with biomicroscopy. Thorough explanation about the study was done for all patients, and informed consent was obtained from them.

Scanning with Topcon 3D-2000 OCT (TOPCON Corporation, Tokyo, Japan) was performed using the built-in 3D macula report scan protocol. All study eyes were dilated with mydriatic eye drops before scanning. Intrinsic fixation target was chosen for all eyes, and those who cannot fixate on that target are excluded from the study. One doctor did the scans for all eyes; also, one doctor analyzed all the measurements (AH).

Horizontal scans passing through the foveal center were chosen. The ELM, IS/OS, inner and outer boundary of RPE layers are identified by layer segmentation from the OCT machine; central macular thickness (CMT) is considered as central subfield thickness which was determined from the inner 1 mm circle in the nine ETDRS areas of the retinal thickness map. ORL and PROS thicknesses are manually measured with caliber identification in the machine. The ORL thickness is defined as the distance between the ELM and the outer border of the RPE, while the PROS length is defined as the distance between the inner border of the IS/OS junction and the inner border of the RPE.

### 2.1. Statistics

The measurements for all eyes are input in excel sheet for statistical analysis. All parameters are expressed in mean with standard deviation, and the mean value comparison between the two groups regarding ORL, PROS, CMT, and BCVA was calculated using the unpaired *t* test. BCVA was converted to logMAR for statistical analysis. In group ΙΙ, correlations between CMT, ORL, and PROS with BCVA were done using Pearson's correlation formula. The IBM SPSS Statistics (v 15.0; IBM Corporation, Chicago, IL) was used. *p* values ≤0.05 were accepted as being statistically significant.

## 3. Results

The study included 80 eyes that were divided into two groups, group Ι (40 eyes) and group ΙΙ (40 eyes). The demographic data of both groups are summarized in Table 1. The mean logMAR BCVA was 0.1 in group Ι, while it was 0.4 ± 0.2 (ranging from 0.3 to 1.00) in group ΙΙ (SS, *p* ≤ 0.05).

### 3.1. CMT, ORL, and PROS Thicknesses in Both Groups

**Figure 1:**
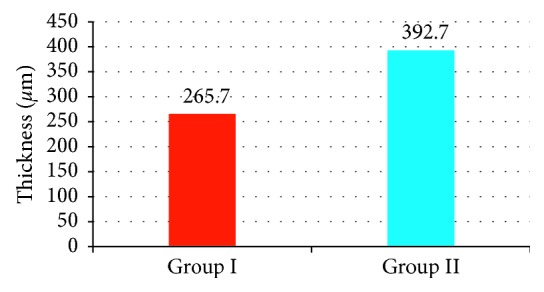
The mean central macular thickness in both groups.

**Figure 2:**
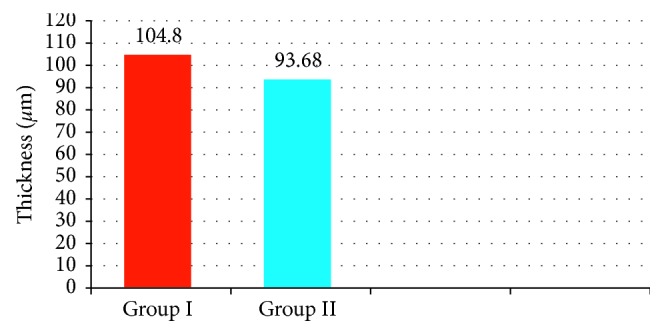
The mean ORL thickness in both groups.

**Figure 3:**
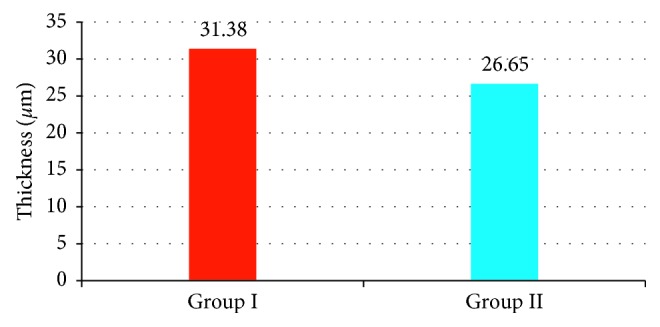
The mean PROS length in both groups.

**Figure 4:**
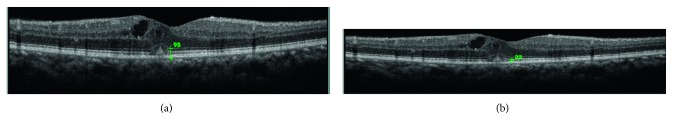
Diabetic patient with BCVA 20/50 (0.4 logMAR). Central subfield thickness is 354 *µ*m, ORL thickness is 95 *µ*m, and PROS length is 22 *µ*m.

**Figure 5:**
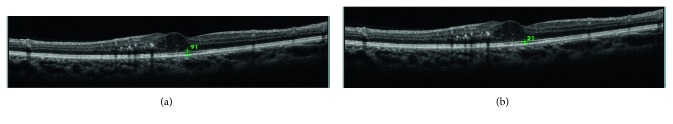
Diabetic patient with BCVA 20/60 (0.5 logMAR). Central subfield thickness is 378 *µ*m, ORL thickness is 91 *µ*m, and PROS length is 21 *µ*m.

CMT was statistically significant higher in group ΙΙ than group Ι (392.70 ± 62.91 *µ*m and 265.73 ± 17.17 *µ*m, respectively) (*p* < 0.001) as shown in Figure 1. ORL thickness was statistically significant higher in group Ι than ΙΙ (104.80 ± 4.94 *µ*m and 93.68 ± 6.34 *µ*m, respectively, *p* < 0.001) as shown in Figure 2. Regarding PROS length, it was statistically significant higher in group Ι than group ΙΙ (31.38 ± 3.4 *µ*m and 26.65 ± 3.39 *µ*m, respectively, *p* < 0.001) as shown in Figure 3. Figures 4 and 5 are examples of patients in group ΙΙ with their corresponding ORL thickness and PROS length.

### 3.2. Correlation between BCVA and Different OCT Parameters

**Figure 6:**
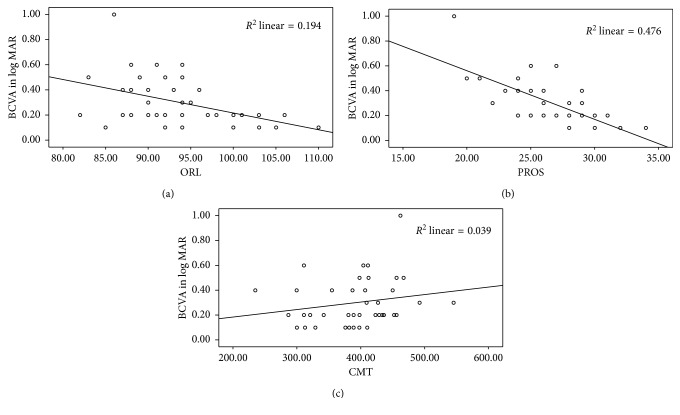
Correlation between BCVA and ORL thickness, PROS length, and CMT, respectively.

As shown in Figure 6(a), there was statistically significant moderate correlation between logMAR BCVA and ORL thickness with correlation coefficient (*r*) = −0.440 and square of correlation coefficient (*r*^2^) = 0.194 with *p* value = 0.004. There was also strong correlation between logMAR BCVA and PROS length in group ΙΙ (*r* = −0.690, *r*^2^ = 0.476, with *p* value < 0.001), as shown in Figure 6(b), whereas there was statistically insignificant weak correlation between logMAR BCVA and CMT (*r* = 0.198 with *p*=0.220) as shown in Figure 6(c).

## 4. Discussion

DME is one of the most important and commonly encountered macular diseases that can lead to significant drop of vision. Long-standing DME can lead to different pathological retinal abnormalities in addition to other clinical abnormalities in visual acuity, visual field, microperimetry, and dark adaptation. OCT has been found to be an important and reliable diagnostic and prognostic modality for retinal diseases. The new generations of the OCT machines with their high resolution can identify retinal microstructures as ELM, IS/OS junction, and RPE layers. CMT is the most commonly used OCT parameter for assessment of DME, but it has been reported in some studies that it could not be correlated with BCVA [4–7, 12, 13].

This study observed statistically significant higher PROS length in group Ι than group ΙΙ (31.38 ± 3.4 and 26.65 ± 3.39, respectively), which is the same as Ozkaya et al. [8] showed in their study (32.6 ± 6.9 for DME eyes and 38.5 ± 2.5 for healthy eyes); also Verma et al. [14] showed that photoreceptor layer thickness was significantly less in patients with DM than healthy eyes (68.79 ± 7.84 *µ*m and 61.62 ± 4.48 in diabetes). The explanation of thinner foveal PROS length in DME may be attributed to hypoxia resulting from relative outer retinal layer hypoperfusion in addition to increased demand of oxygen resulting from the high metabolic rate in this area.

ORL is the distance between ELM and RPE which is the length of both inner and outer segment of the photoreceptor layer. The mean ORL thickness in this study was significantly greater in group Ι than that in group ΙΙ (104.80 ± 4.94, 93.68 ± 6.34, *p* < 0.001) which is also the same as Eliwa et al. [15] reported in their study (99.9 *µ*m in normal eyes versus 83.3 *µ*m in DME). The explanation for this finding may be related to relative decrease in choroidal blood flow and resultant choroidal ischemia.

There was strong positive correlation between BCVA and PROS length in group ΙΙ (*r* = 0.690, *p* value < 0.001) which is comparable to Alasil et al. [16], Ozkaya et al. [8], and Forooghian et al. [11]. It is proved that long-standing DME can result in damage to the photoreceptors with its outer segment being reduced in length than the average length in normal eyes. As the outer segments contain opsin which is essential to signal transduction, vision would be affected if there is significant decrease in outer segment thickness.

In this study, there was moderate correlation between the BCVA and ORL thickness that was statistically significant and was similar to Eliwa et al. [15] and Wong et al. [17]. The inner segment is also important for the normal function of the photoreceptor cell layer as it contains the ATP which is the source of energy. Since both inner and outer segments are important for visual processing, any affection of the thickness of the outer and inner segments would affect the visual functions.

The study showed that BCVA is correlated more with PROS length than ORL thickness in DME patients which may give us the impression that PROS length is a more sensitive parameter to be used as the predictor of vision in these patients; this observation may be explained by the speculation that the role of the outer segment as opsin reservoir is more important than the role of the inner segment as the ATP source; however, further studies are needed to discuss this issue.

On the other hand, there was weak correlation between BCVA and CMT (*r* = −0.198); this demonstrates good agreement between the findings of our study and Forooghian et al.'s study [11]. The same finding was found in one of diabetic retinopathy clinical research network study [6]. Actually, studying the thickness of PROS and ORL would be more important than just CMT as the initial and prognostic factor for DME patients because edema, which is just accumulation of fluid, could resolve with time and appropriate treatment and hence, the CMT would be less, but the status of integrity and thickness of the photoreceptors will persist even after resolution of DME; however Mori et al. [18], showed significant restoration of the IS/OS and ELM after intravitreal injection of ranibezumab, which may highlight that anti-VEGF treatment blocks neurotoxic blood constituents and allows restoration of the inner and outer segments from the surviving cell bodies of the photoreceptor cells in the ONL.

Limitations of this study include absence of DME patients with disrupted ELM, IS/OS junction, and eyes with serous foveal detachment, so the results of these study cannot be applied to these patients; also, measurement of the ORL and PROS was done manually in the foveal center which is time consuming and may create some errors in determination of these measurements.

In conclusion, the present study showed that DME can lead to reduced thickness of ORL and PROS. Thickness of both ORL and PROS correlates better with visual acuity than CMT in DME patients. It would be reasonable to consider these variables as initial and prognostic factors for DME patients.

## Figures and Tables

**Table 1:** The different demographic characters of both groups.

		Group Ι		Group ΙΙ		Test of significance	
		Mean (*N*)	SD (%)	Mean (*N*)	SD (%)	*p* value	Significance
Age		59.70	6.94	59.83	7.23	0.937	NS

Duration of DM in group ΙΙ				14.43 years	3.51		

Sex	Male	21	52.5	24	60.0	0.499	NS
	Female	19	47.5	16	40.0	0.678	

Eye	OD	20	50	22	55.0	0.823	NS
	OS	20	50	18	45.0	0.956	

## Data Availability

All the data used to support the findings of this study are available from the corresponding author upon request.
